# The relationship between patient deaths in hospital wards and medical students’ emotional distress: evidence from two newly established medical schools in Zimbabwe

**DOI:** 10.3389/fpsyt.2025.1655790

**Published:** 2025-10-20

**Authors:** Mqemane Tshababa, Regis Chireshe, Julia Mutambara, Mokoena Patronella Maepa

**Affiliations:** ^1^ Clinical Psychology, Sefako Makgatho Health Sciences University, School of Medicine, Pretoria, South Africa; ^2^ Special Needs Education, Great Zimbabwe University, Masvingo, Zimbabwe; ^3^ Psychiatry, Midlands State University, Gweru, Zimbabwe

**Keywords:** coping, exposure, distress, dying patients, medical students, psychological impact

## Abstract

Patient deaths in hospital wards can have serious emotional and psychological impacts on medical students in the clinical training phase. While the literature reports various levels of distress caused by patient deaths among medical students globally, there is no study on the relationship between patient deaths and medical students’ distress at two newly established medical schools in Zimbabwe. Therefore, this study sought to determine the relationship between patient deaths and medical students’ distress levels. A positivist research philosophy, which utilizes a quantitative approach, was adopted for the study. Gadzella’s (2005) Revised Student-Life Stress Inventory was used to determine the sources and to measure the levels of distress among medical students. A sample of 123 medical students drawn from two newly established medical schools in Zimbabwe participated in the present study. Of these participants, 65 were men (52.8%) and 58 were women (47.2%). The mean age of the participants was 23.4 years. The results indicated significant variations in distress among the different educational levels of medical students at the *p* < 0.05 level for the five conditions (*F* = 139.95, *p* = 0.000). The results of the study revealed that exposure of medical students to dying patients was positively correlated (*β* = 0.211) with their distress. In addition, the results on exposure to dying patients explained a significant amount of variance in distress (*F* = 23.519, *p* = 0.000, *R* ^2^ = 0.189, *R* ^2^adjusted = 0.181). A correlation coefficient of *R* = 0.435 was found, indicating a moderate positive linear relationship between patient deaths and medical students’ distress. The findings of this study highlight that medical students experience emotional distress due to exposure to dying patients. It is recommended that medical students are provided training on emotional resilience and end-of-life care to enable them to handle patient deaths in hospital wards.

## Introduction

Medical students are vulnerable to a variety of stressors during their medical training. Some potential stressors for students undergoing medical training include the following: the nature of the training curriculum itself, the number of years taken by medical students to finish their training program, the number of hours they spend in school studying, and the financial distress that comes with many years of schooling. The extended period of learning and exposure to patient deaths during the clinical years of training are among other potential stressors (1, 2). For medical students, the experience of witnessing patient death is generally associated with negative mental health experiences. Patient deaths in hospital wards are not uncommon. Wilson et al. (3) reported that 167,464 of alladmitted patients in Canada die per year (58.8%), 13,617 of all admitted patients in Norway die per year (32.5%), 22,532 of all admitted patients in Switzerland die per year (38.4%), 428,753 patients in Germany die per year (45.64%), and 813,249 of all admitted patients in the United State of America die per year (28.5%). In South Africa, a study reported that 1,701 of the admitted patients at one hospital died during hospitalization, representing a death rate of 15.1%. In Zimbabwe, the study by Mujuru and Kambarami (4) revealed that deaths in hospital wards, particularly in pediatric wards, are a common sight for physicians. In their study, the authors found that, out of 155 children admitted in a Zimbabwean hospital, 21% of these patients die within 24 h of admission. The aforementioned statistics highlight the inevitability of deaths in hospital wards, and as such, medical students so exposed might experience psychological distress emanating from such experiences.

Studies such as those of Burns (5)Baranauskas et al. (6), and Mendes et al. (7) also examined how medical students respond to witnessing patient death and have consistently highlighted the complex interplay between emotional distress, resilience, and institutional support. Similarly, a cross-sectional study conducted in New Zealand by Heath et al. (8) on palliative and end-of-life care among undergraduate medical students found that the experience of seeing a patient dying brought them emotional distress. This Kiwi study found that the dilemma faced by some medical students was how to balance being able to protect themselves by becoming emotionally detached, yet still being able to display empathy and care for their patients who may after all not win the battle against the disease.

In exploring how medical students perceive and navigate encounters with dying patients, Smith-Han et al. (9) identified three major themes related to death and dying: a) students’ reactions to death and their means of coping; b) changing perceptions about the role of a physician, the practice of medicine, and personal identity; and c) the professional environment, roles, and responsibilities. The study by Fernandez-Avalos et al. (10) highlighted that witnessing the death of a patient can make medical students experience reduced empathy to cope with emotional anguish, and this may lead them to seek reassurance through the solace of colleagues. Medical students also change their perceptions, wherein they initially see themselves as curative heroes to carers who realized that patient death is an inherent component of life and is not something traumatic. In view of the above, patient deaths in clinical settings can also be construed to help medical students grasp the routine nature of mortality, understand procedural responsibilities, and develop a sense of accountability (8).

A narrative literature review by Lisai-Goldstein and Shaulov (11) found that exposure to dying patients evokes previous experiences of bereavement and causes emotional distress among students. Their findings further revealed that medical student experience emotional distress (i.e., anxiety, burnout, and related mental health problems). The study also revealed that the majority of patients receive much of their healthcare toward the end of life; hence, medical students in their clinical years are frequently confronted with issues related to death and dying for the first time. Despite the available evidence of distress resulting from end-of-life crisis, the medical school curriculum often focuses exclusively on disease diagnosis and treatment and pays little attention to education about end-of-life issues and palliative care. Dyrbye et al. (12) further showed the frequency with which students encounter patients at the end of life and the lack of student training in this area; hence, it is no surprise that students are fearful, anxious, and hesitant to interact with dying patients. Medical practitioners generally report feeling awkward, sad, overwhelmed, apprehensive, vulnerable, angry, and anxious in these circumstances, which highlights the lack of an adaptive coping model that medical students and medical practitioners could use and can precipitate thoughts about one’s own death (13). Although medical students undergo a curriculum that prepares them for end-of-life crisis, some of them are still inadequately prepared to deal with patient deaths. One meta-analysis study reported that, although 100% of third-year students had cared for a terminally ill patient, only 41% had been present while an attending physician talked with a dying patient, and only 35% had ever discussed with an attending physician how to care for terminally ill patients, hence lacked the practical strategies to handle distressing situations such as a patient’s death (12).

To examine the link between witnessing dying patients and distress, studies carried out in Brazil by Kushal et al. (14)Neto et al. (15), and Adams and Walls (16) indicated that emotional distress is a potential cause of concern and has severe psychological effects on health professionals, such as medical practitioners. According to Neto et al. (15), emotional distress is characterized by mental issues such as anxiety emanating from various clinical activities and depression in the face of the coexistence of countless deaths.

In the existing body of literature, several other studies have examined the association between patient deaths and emotional distress among medical students, concluding that no significant correlation is found between these variables. For example, a Canadian study by Davies (17) presupposes that attending to death and dying patients in hospital wards is a routine activity and is part of the daily experience of medical practitioners. This study concluded that death is not linked to distress in the generality of medical practitioners and that those experiencing distress are normally those who face other work-related challenges such as disciplinary hearing and/or financial complications. In support, a study conducted in Turkey by Akyol et al. (18) posits that exposure to patient deaths is not inherently stressful to all medical practitioners, but that it can be stressful to those who are not prepared for the emotional experiences that come with the death of patients while under their care.

Closer to home, in South Africa, Van de Venter (19)Snyders (20), and Dove et al. (21) examined the association between patient deaths and distress among undergraduate students undergoing medical training. These studies found that students at the clinical phase of their medical training experience distress due to exposure to dying patients and death. In a Zimbabwean context, there is limited literature on patient deaths and medical students’ distress. A search of the literature uncovered one study that examined medical students’ distress. This study focused on the link between personal life events and medical students’ distress (22), and it was found that losing a loved one, falling sick, or having a sick member in the family are also contributors to medical students’ distress.

While it is not the responsibility of the students to care for the most seriously ill patients in hospitals and clinics, the inadequate number of qualified medical practitioners in Zimbabwe appears to be pushing medical students to the front end of medical care. As a result, medical students are generally exposed to more dying patients. Witnessing illness or death during the performance of duty may lead to negative health outcomes for other patients due to the distressed nature of the medical practitioners themselves. Hence, the present study sought to determine the relationship between patient deaths and medical students’ distress and to propose measures that can help medical students manage emotional distress.

This study is based on Lazarus and Folkman’s (23) Transactional Model of Stress and Coping. This model emphasizes the threatening tendency of stress and posits that how one appraises the stressor contributes to their stress or lack of it. The model highlights that it is not only about the event being perceived that causes distress but also how the individual interprets the event is key to distress. According to this model, if an individual interprets death in hospital wards as one of the stages of human development, he/she will not feel distressed; however, if one interprets death as a permanent loss, he/she will feel distressed. Anchoring this study on this transactional model helps in the design of interventions for coping. The model suggests three coping strategies that inform interventions: problem-focused coping, emotion-focused coping, and the potentially maladaptive emotion-focused coping. Problem-focused coping implies that an individual identifies the source of distress or the stressor and then carries out an action that helps him or her avoid or reduce the effects of the stressor, and it includes dimensions such as active coping and planning (24). On the other hand, emotion-focused coping is concerned with bypassing the stressor and focusing attention on managing feelings, which cause the distress. Potentially maladaptive emotion-focused coping entails denial, mental disengagement, behavioral disengagement, the use of alcohol and drugs, and focusing on and venting of emotions (24).

Medical students, especially those who are in their clinical years, are vulnerable to emotional distress when they witness patients dying in hospital wards. Medical schools should provide a supportive learning environment that promotes emotional and psychological wellbeing among medical students (22). However, evidence from the literature suggests that medical students in Zimbabwe experience emotional distress due to patient deaths and that this affects their mental health, their academic performance, and their future as health practitioners. This study applied a survey design to understand the relationship between patient deaths and medical students’ distress, which could help in the development of interventions that foster resilience and wellbeing in medical students. The objective of this study was to determine whether there is a significant relationship between patient deaths and medical students’ distress at two newly established medical schools in Zimbabwe.

## Methods

### Study setting

The study was carried out at two newly established medical schools in Zimbabwe, which were established within the last 20 years, and are situated in two state universities in Zimbabwe. Both medical schools offer Bachelor of Medicine and Bachelor of Surgery degree programs over a period of 6 years. In this study, these two medical schools were anonymized as Medical School A and Medical School B.

### Participants

A total of 123 medical students drawn from the two newly established medical schools in Zimbabwe took part in the study. To be eligible for participation in this study, one needed to be a medical student at either Medical School A or Medical School B, these being the two newly established medical schools in Zimbabwe. Furthermore, the respondents must have witnessed patients dying in the hospital wards before the commencement of this study. Those eligible for the study were then chosen to participate using the stratified sampling technique and the simple random technique.

### Instrument

#### Gadzella’s 2005 revised Student-Life Stress Inventory

Gadzella’s (25) revised Student-Life Stress Inventory questionnaire was used to determine the sources of distress of medical students. The Gadzella Scale was chosen primarily for its strong psychometric properties. It has a high internal consistency (*α* = 0.93 overall) that allows researchers to obtain enhanced reliability and validity. The scale is a revision of the original Student-Life Scale Inventory (25), which is a 51-item Likert-type scale. The Revised Student-Life Stress Scale used in this study has 21 items, each rated from 1 to 5. In this scale, 1 indicates that the identified stressor does not affect the student at all, while a rating of 5 indicates that the identified stressor affects the student very much. The Gadzella Scale includes items such as those that ask about the academic life of students, e.g., the demands of school work, conflicts with deans, and other personal life issues such as the loss of a loved one, sickness of a family member, and patient deaths. Using this scale, respondents who obtained a score within 84–105 were considered to have severe emotional distress, those with a score 63–84 reflected moderate emotional distress, and those who obtained a score of 42–63 reflected mild emotional distress.

### Procedures

The researchers distributed 123 structured questionnaires to the participating medical students by hand. The questionnaires included a demographic section, which asked students about their age range, gender, year of study, relationship status, and ethnicity. The questionnaires also included a main section comprising the Likert scale-type statements that rated the responses from 1 to 5 depending on whether they agreed or disagreed with the scale. On this scale, 1 represents strongly disagree, while 5 represents strongly agree. The respondents were given 3 days to complete the questionnaires, and the researchers physically collected the completed questionnaires.

### Data analysis

The IBM SPSS version 28 was used to explore the correlations between exposure to patient deaths and medical students’ emotional distress. Linear regression analysis was used to examine the link between medical students’ exposure to dying patients and their distress. Linear regression analysis was performed to determine whether there is a positive or a negative relationship between exposure to death and the levels of distress among medical students. A positive relationship means that a higher exposure to patient deaths is linked to higher distress, while a negative relationship means that more exposure to death is associated with lower levels of distress. Analysis of variance (ANOVA) was used to determine whether there are significant differences in the emotional distress levels among medical students in different levels of the medical program.

### Data storage

The collected data are stored in an encrypted external hard drive and shall be so stored for a duration of 5 years, in line with ethical guidelines from the Medical Research Council of Zimbabwe.

### Ethical clearance

The Medical Research Council of Zimbabwe approved this study (clearance no. MRCZ/A/2798). All participants signed an informed consent form to indicate their willingness to participate in the study. The participants were informed of the principles of beneficence and non-maleficence. The researchers explained how this study would benefit medical students and the greater society. The respondents were also informed that no harm was intended in this study and that they were therefore free to continue or discontinue the study at any stage of the research. The respondents voluntarily participated in this study, and no incentives were offered. Those who opted not to participate were assured that their non-participation is not going to affect their progression in the program. Those respondents who displayed negative emotional responses were referred to the respective medical school’s Department of Psychiatry, Social and Behavioral Sciences for counseling and therapeutic services.

## Results

### Demographic information

**Table 1** shows the various biographical variables examined in the study to determine the extent to which these stress factors were able to influence the strength and the direction of the relationships between variables.

**Table 1:** Biographical data (*n* = 123).

Biographical variable	Biographical description	Frequency	Percentage
Gender	Men	65	52.8
	Women	58	47.2
Age	18–22 years	70	57
	23–27 years	51	41
	28–32 years	01	0.8
	33–50 years	01	0.8
Relationships status	Single and dating	66	53.6
	Single and not dating	56	45.5
	Married	1	0.8
Academic year	First year	21	17.0
	Second year	32	26.0
	Third year	28	22.8
	Fourth year	18	14.6
	Fifth year	24	19.5
Residence	On campus	114	92.7
	Other	9	7.3
Educational qualification of parents	O level	35	28.4
	Diploma	22	17.9
	Degree	12	9.8
	Post-Graduate	11	8.9
	Other	43	35.0
Nature of parents’ degree	Medicine	9	7.3
	Engineering	1	0.8
	Social Sciences	13	10.6
	Other	86	70.0
	No response	14	11.3
Nature of siblings’ degree	Engineering	3	2.4
	Social Sciences	17	13.8
	Other	75	61.0
	No response	28	22.8
Family income status per month ZWG	1–20,000	16	13.0
	21,000–40,000	34	27.6
	41,000–60,000	10	8.1
	61,000 and over	56	45.5
	No response	7	5.7
Ethnicity	African	122	99.2
	European	1	0.8
	Asian	0	0
Religion	Christianity	113	91.8
	Muslim	8	6.5
	No response	2	1.6

### Descriptive statistics

**Table 2** shows the emotional impact of patient deaths, with a mean score of 2.54, a high skewness of 1.660, and kurtosis of 2.629. Academic pressure, on the other hand, had a mean of 1.93 and a lower skewness of 0.663, which appeared less distressing compared with the emotional impact of patient deaths. Also of interest was that personal health concerns showed a mean of 2.29 and a skewness of 0.267, implying higher distress levels.

**Table 2:** Sources of medical students’ distress.

Stress factor	n	Mean	Standard deviation	Skewness	Kurtosis
Conflicts in school	109	1.79	0.771	1.373	3.904
Academic pressure	69	1.93	1.204	0.663	−1.320
Study avoidance *vs*. social needs (procrastination tension)	109	1.95	1.265	1.598	1.481
Major life changes	100	2.05	0.770	0.996	2.797
Social isolation	109	2.14	0.887	0.861	1.382
Campus culture misalignment	109	2.17	0.731	0.586	1.357
Difficulty balancing academics and personal life	109	2.24	0.952	0.750	0.268
Overwhelming academic workload	100	2.25	0.947	0.861	0.465
Religious dissonance	109	2.26	0.985	0.231	−0.736
Emotional impact of patient death	100	2.54	0.846	1.660	2.629
Unsatisfactory living state	109	2.30	0.752	1.034	2.931
Negative self-perception	109	2.33	0.933	0.613	0.129
Family-related stress	109	2.37	0.868	0.593	1.335
Perceived racial or ethnic bias	109	2.37	1.220	0.786	−0.004
Anxiety on career decisions	99	2.42	1.238	1.413	0.611
Relationship strain	109	2.51	0.801	0.230	1.275
Personal health concerns	109	2.29	0.864	0.267	0.325
Substance use issues	109	2.75	1.081	0.556	−0.137
Illness or health concerns of loved ones	109	2.76	0.781	0.686	0.980

Linear regression analysis examined the link between medical students’ exposure to dying patients and their distress. **Table 3** reveals the findings.

The aim of this study was to determine whether there is a significant association between patient deaths and medical students’ distress. Linear regression analysis examined the association between medical students’ exposure to dying patients and their emotional distress. **Table 3** reveals that medical students’ exposure to dying patients and their emotional distress were positively correlated (*β* = 0.211). In addition, the results on exposure to dying patients and emotional distress explained a significant amount of variance in distress (*F* = 23.519, *p* = 0.000, *R* ^2^ = 0.189, *R* ^2^adjusted = 0.181).

**Table 3:** Linear regression for medical students’ exposure to dying patients *versus* distress.

Unstandardized coefficients			Standardized coefficients	*T*	Sig.	Collinearity statistics	
Model	*B*	SE	*β*			Tolerance	VIF
(Constant)	30.231	3.176		11.135	0.000		
Death	0.723	0.651	0.211	2.234	0.022	1.000	1.000
Regression equation statistics							
*F*			23.519				
*P*-value of the *F* statistic			0.000				
*R*2			0.189				
*R* ^2adjusted^			0.181				

*SE*, standard error; *VIF*, variance inflation factor.

a Dependent variable: distress.

^b^Predictor: (Constant), death.

**Table 4** shows how exposure to death significantly predicts emotional distress in medical students, but it explains only a modest portion of the variance. The model is statistically sound, suggesting other variables also contribute to medical students’ distress.

**Table 4:** Model summary^a^ of medical students’ exposure to death and their emotional distress.

Model	*R*	*R* ^2^	Adjusted *R* ^2^	SE of the estimate	Change statistics		df1	df2	Sig. *F* change	Durbin–Watson
					*R* ^2^ change	*F* change				
1	0.435^b^	0.189	0.181	0.51179	0.189	23.519	1	101	0.000	1.961

a Dependent variable: distress.

b Predictor: (Constant), death.

**Table 5** indicates a statistically significant relationship between medical students’ exposure to death and their emotional distress. The regression model explains a meaningful portion of the variance in distress scores (F = 23.519, p < 0.001), suggesting that exposure to death is a strong predictor of emotional distress in this sample.

**Table 5:** ANOVA^a^ for medical students’ exposure to death and their emotional distress.

Model		Sum of squares	*df*	Mean square	*F*	Sig.
1	Regression	6.160	1	6.160	23.519	0.000^b^
	Residual	26.455	101	0.262		
	Total	32.615	102			

a Dependent variable: distress.

b Predictor: (Constant), death.

This study revealed that there is a moderate positive relationship between exposure to dying patients and distress, with a correlation coefficient of *R* = 0.435. The goodness of fit of the observed data was 18.9%, suggesting that while the result showed a meaningful relationship, it also showed that other factors, such as personal life events, the academic workload, and the financial status of medical students, also contributed to their distress. In terms of the ANOVA, the results indicated an *F*-statistic of 23.519 (*p* = 0.000). This examined whether patient death significantly explains the variance in distress. As the *p*-value was 0.000, it is therefore indicated that exposure to patient deaths significantly predicts emotional distress. Simply put, the above results showed that there is a statistically significant relationship between exposure to patients dying and medical students’ emotional distress. It also showed that while exposure to patient deaths constituted 18.9% of the variance in distress, other factors such as the academic workload, the financial status, and students’ personal life events also contributed to their distress.

## Discussion

The objective of this study was to determine whether there is an association between patient deaths and medical students’ distress. The study established that there is a significant association between patient deaths and medical students’ distress. In terms of demographic information, only gender, age, and the academic level of the students appeared to have influenced the results of the study. In the present study, female medical students reported higher levels of distress compared with their male counterparts. This finding confirms those of the study by Mommersteeg et al. (26), which found that women experienced more distress than did men. The present study found that the age and the academic level of participants were positively correlated with the distress of medical students, which confirmed the findings of Mallaram et al. (27), who reported that the distress among medical students increased in the first year of medical school. The finding that exposure to patient deaths in hospital wards is correlated with emotional distress was confirmed by Smith-Han et al. (28), Dyrbye et al. (12)Neto et al. (15), and Adams and Walls (16). These studies also concluded that, for medical students, witnessing death while visiting hospital wards during the clinical period is a stressor on its own. However, the finding in this study that medical practitioners/medical students witnessing the death and dying of patients is linked to their emotional distress contradicts the findings in a Canadian study by Davies (17), which concluded that witnessing the death and dying of patients has no direct link to medical students’ distress. The contradictory findings between this study and that by Davies (17) are possibly due to the nature of the samples used. In his study, Davies (17) used a Canadian sample, which possibly believes in the inevitability of death; hence, the participants could not have implicated death as a stressor as they believe one should be psychological prepared for it. On witnessing dying patients in hospital wards, the Canadian sample would probably conclude that the inevitable has happened. On the other hand, Zimbabwean medical students, while they do understand that death and dying are part of their experiences as medical students, may be ill-prepared to appreciate and integrate the theory learned in class and the actual practice of medicine. This unpreparedness to understand and accept the death of patients may also be linked to the bonds that have been formed between the medical students and the patients during hospital care. In the event that a patient dies, these bonds and the medical students’ failure to accept patient death may trigger negative emotional responses, thus contributing to their emotional distress.

The studies by Smith-Han et al. (9) and Fernandez-Avalos et al. (10) have important methodological implications for the current study. Both studies used grounded theory as their research design, which is a complete departure from the current study. While the two cited studies had strength of dependability due to the length of the period within which the data were collected, the common weakness of grounded theory was noted; that is, grounded theory methods tend to produce large amounts of data, which are often difficult to manage, and this can lead to inaccurate results. To address the weaknesses of grounded theory noted in the literature, the present study adopted a survey design, which used questionnaires to collect the data. As previously mentioned, the use of a quantitative method helps in obtaining data, thus producing more reliable and valid results. Furthermore, as the studies by Smith-Han et al. (9) and Fernández-Ávalos et al. (10) used grounded theory, their sample sizes were relatively smaller, and researchers agree that the use of small sample sizes makes it impossible to form widespread generalizations around the findings of the study. The present quantitative study used a relatively bigger sample of 148 participants to enable generalization of the findings.

The present study established that medical students’ exposure to dying patients has a moderate yet statistically significant impact on their distress levels. However, as the *R* ^2^ value was not very high, other factors such as personal coping mechanisms, personal life events of the medical students, their financial status, and the academic workload likely contributed to their emotional responses (29). What exacerbates medical students’ distress levels is their belief that their role is to save lives; therefore, if a patient dies while they are professionally helping the client, feelings of professional inadequacy are evoked, hence creating a sense of guilt and anxiety, leading to distress (30).

The finding in the present that patient deaths in hospital wards contributes to medical students’ emotional distress agrees with the findings of Ramsland (31), who found that the manner of death causes uncertainty and anxiety among medical practitioners, particularly if the manner of death is not natural. Generally, there are five commonly accepted manners of death. As mentioned, medical students are frequently exposed to various manners of death, including natural causes, accidental death, suicide, homicide, and death of undetermined cause (32). In the course of their training, they may be confronted with profoundly distressing scenes, such as the body of a person crushed beyond recognition, placing them at risk of emotional and psychological strain (33). It is not uncommon for a student to witness multiple fatalities in a single day, some resulting from harrowing circumstances such as train accidents, hangings, or poisoning. These repeated exposures to such traumatic imagery, particularly for students who lack prior coping mechanisms, can lead to vicarious trauma, a phenomenon where individuals develop trauma symptoms indirectly through empathic engagement with those affected by the trauma (34). Left unaddressed, this cumulative emotional toll may impact students’ mental wellbeing, academic performance, and long-term professional resilience. Facing the reality of death in hospital beds and recognizing its inevitability can trigger feelings of confusion, disorientation, and emotional numbness among medical students who offer care-giving services, and this can lead to them experiencing feelings of profound sadness, despair, anxiety, fear, or intense anger (35). The level of clinical exposure might have influenced the results of this study, where students in the last year of their training exhibited more distress due to their level of attachment as compared with their counterparts who were in the early years of training and had not witnessed numerous deaths during their studies. Similarly, gender and cultural background might have also contributed due to the differences in the emotional expression and coping strategies used by men and women.

This study reinforces the importance of integrating emotional resilience training and palliative care education into the medical curriculum to emotionally and psychologically prepare medical students for distress-evoking situations such as witnessing patient deaths (36). Providing students with coping mechanisms and peer support could mitigate distress and improve their ability to handle end-of-life care compassionately (37). The findings further highlight the importance of including mentorship and role modeling, where senior medical practitioners are encouraged to share their experiences with medical students to prepare them for encounters with mortalities in hospital wards. The strength of the present study is its use of Gadzella’s (25) Student Life-Stress Inventory, which has high internal consistency, making it possible to accurately assess the unique stressors medical students go through during training. The limitation of the present study is that, due to its quantitative nature, it did not capture the lived experiences of the medical students. Capturing the lived experiences of medical students would have shed more light on the exact nature of distress they have experienced.

It is recommended that medical students be trained on peer-to-peer counseling so that they can provide psychological first aid to each other during the initial stages of distress and while awaiting the arrival of qualified mental health practitioners. Peer-to-peer counseling will also help students adopt adaptive stress management techniques, as opposed to maladaptive strategies that lack sustainability. It is important to scale up the teaching of death education and emotional resilience to fully prepare medical students for the emotional toll that comes with patient deaths in hospital wards.

## Conclusion

In this study, exposure to death is a significant predictor of emotional distress. The ANOVA results confirmed that the relationship between exposure to dying patients and emotional distress is statistically significant, indicating that there is a very low probability that this correlation is due to chance. The predictor variable (death) has a meaningful impact on the dependent variable (distress). Additional factors such the academic workload, difficulty in balancing academics and personal life events, and personal health concerns also played a role in medical students’ emotional distress. While patient death is part of medical students’ experiences, it is important for the medical fraternity to help these students handle the pressure that comes with their work environment.

## Data Availability

The original contributions presented in the study are included in the article/supplementary material. Further inquiries can be directed to the corresponding author.

## References

[1] Santiago IS de Castro e Castro S de Brito APA Sanches D Quintanilha LF Avena KDM. Stress and exhaustion among medical students: a prospective longitudinal study on the impact of the assessment period on medical education. BMC Med Educ. (2024) 24:630. doi: 10.1186/s12909-024-05617-6, PMID: 38844948 PMC11154982

[2] Ebrahim OS Sayed HA Rabei S Hegazy N. Perceived stress and anxiety among medical students at Helwan University: A cross-sectional study. J Public Health Res. (2024) 13:22799036241227891. doi: 10.1177/22799036241227891, PMID: 38313630 PMC10838489

[3] Wilson DM Fabris LG Martins AL Dou Q Errasti-Ibarrondo B Bykowski KA. Location of death in developed countries: are hospitals a primary place of death and dying now? OMEGA-Journal Death Dying. (2025) 91:781–97., PMID: 36475942 10.1177/00302228221142430 PMC12018720

[4] Mujuru HA Kambarami RA. Mortality within 24 hours of admission to the Paediatric Unit, Harare Central Hospital, Zimbabwe. Cent Afr J Med. (2012) 58:17–22., PMID: 26255330

[5] Burns RB. Managing grief and death. In: The Human Impact of the COVID-19 Pandemic: A Review of International Research. Springer Nature Singapore, Singapore (2023). p. 273–96.

[6] Baranauskas M Kalpokas M Kupčiūnaitė I Lieponienė J Stukas R. Self- perceived stress in association with emotional experiences following patient death and coping adequacy among clinical nurses in Lithuania: A cross-sectional study. J Clin Med. (2024) 13:2533. doi: 10.3390/jcm13092533, PMID: 38731062 PMC11084392

[7] Mendes TDMC Vasconcelos HSD Oliveira NPDD Souza DLBD Castro JLD. Impact of work on the mental health and coping strategies of the oncological hospital multiprofessional team: systematic literature review. Rev Bras Cancerol. (2025) 70:e-244853.

[8] Heath L Egan R Losua E Walker R Ross J MacLeod R. Palliative and end of life care in undergraduate medical education: a survey of New Zealand medical schools. BMC Med Educ. (2022) 22:530. doi: 10.1186/s12909-022-03593-3, PMID: 35804380 PMC9264288

[9] Smith-Han K Martyn H Barrett A Nicholson H. That’s not what you expect to do as a doctor, you know, you don’t expect your patients to die.” Death as a learning experience for undergraduate medical students. BMC Med Educ. (2016) 16:108–20. doi: 10.1186/s12909-016-0631-3, PMID: 27080014 PMC4832523

[10] Fernández-Ávalos MI Fernández-Alcántara M Cruz-Quintana F Turnbull OH Ferrer- Cascales R Pérez-Marfil MN. Coping with death and bereavement: A proactive intervention program for adults with intellectual disability. J Ment Health Res Intellect Disabil. (2023) 3:1–24. doi: 10.1080/19315864.2023.2169420

[11] Lisai-Goldstein Y Shaulov A. Medical students’ Experience of a patient’s death and their coping strategies: A narrative literature review. Am J Hospice Palliative Medicine®. (2025) 42:413–20. doi: 10.1177/10499091241264523, PMID: 38906091 PMC11869511

[12] Dyrbye LN Thomas MR Shanafelt TD. Systematic review of depression, anxiety, and other indicators of psychological distress among Canadian medical students and US. Acad Med. (2006) 81:354–73. doi: 10.1097/00001888-200604000-00009, PMID: 16565188

[13] Haardt V Cambriel A Hubert S Tran M Bruel C Philippart F. General practitioner residents and patients end-of life: involvement and consequences. BMC Med Ethics. (2022) 23:1–11. doi: 10.1186/s12910-022-00867-9, PMID: 36463158 PMC9719227

[14] Kushal A Gupta S Mehta M Singh MM. Study of stress among health care professionals: A systemic review. Int J Res Foundation Hosp Healthc Adm. (2018) 6:6–11. doi: 10.5005/jp-journals-10035-1084

[15] Neto MLR Almeida HG Esmeraldo JDA Nobre CB Pinheiro WR de Oliveira CRT. When health professionals look death in the eye: the mental health of professionals who deal daily with the 2019 coronavirus outbreak. Psychiatry Res. (2020) 18:112–23. doi: 10.1016/j.psychres.2020.112972, PMID: 32302817 PMC7152886

[16] Adams JG Walls RM. Supporting the health care workforce during the COVID- 19 global epidemic. JAMA. (2020) 323:1439–40. doi: 10.1001/jama.2020.3972, PMID: 32163102

[17] Davies M. Death is part of a doctor’s job. BMJ. (2016) 355:16–32. doi: 10.1136/bmj.i5597

[18] Akyol C Celik SU Koc MA Bayindir DS Gocer MA Karakurt B. The impact of patient deaths on general surgeons’ Psychosocial well- being and surgical practices. Front Surg. (2022) 9:1–20. doi: 10.3389/fsurg.2022.898274, PMID: 35574543 PMC9096651

[19] Van de Venter R. Death and dying patients: experiences and coping mechanisms of undergraduate diagnostic radiography students during workplace learning. Capetown: Cape Peninsula University of Technology (2021).

[20] Snyders LL. Experiences and coping mechanisms of young doctors following the death of a patient: a qualitative study. University of Cape town (2021) 2:55–70.

[21] Dove G Botes M Colborne K Lestoalo P Mashiane P Moodley S. An investigation into the well-being of pre-clinical medical students at the University of the Witwatersrand. Undergrad Res Health J. (2023) 1:43–47.

[22] Tshababa M Chireshe R Mutambara J. The Nexus between Personal Life Events of Medical Students and Distress: A Case of Two Newly Established Medical Schools in Zimbabwe Vol. 2. Harare: OIKOS: The Ezekiel Guti University Bulletin of Ecology, Science Technology, Agriculture, Food Systems Review and Advancement (2023).

[23] Folkman S Lazarus RS. Stress processes and depressive symptomatology. Journal of Abnormal Psychology. (1986) 95 (2):107–13. 10.1037//0021-843x.95.2.107 3711433

[24] Senderayi P. Personality as a predictor of job stress among teachers' college lecturers in Zimbabwe. Durban, University of KwaZulu-Natal. (2021).

[25] Gadzella BM Baloglu M Masten WG Wang Q. Evaluation of the student life-stress inventory-revised. Journal of Instructional Psychology. (2012) 39 (2):82–91.

[26] Mommersteeg PM van Valkengoed I Lodder P Juster RP Kupper N. Gender roles and gender norms associated with psychological distress in women and men among the Dutch general population. J Health Psychol. (2024) 29:797–810. doi: 10.1177/13591053231207294, PMID: 37933100 PMC11292987

[27] Mallaram GK Gopalakrishnan U Mathews DA Mudamala DS Gangavarappagari H Modi U. Stress, anxiety, and depression in the first-year students of medical education: A prospective cohort study from a women’s medical college in south India. Indian J psychol Med. (2024), 025371762041282100. doi: 10.1177/02537176241282100, PMID: 39568984 PMC11574819

[28] Smith-Han K Collins E Asil M Blakey AG Anderson L Berryman E Wilkinson TJ. Measuring exposure to bullying and harassment in health professional students in a clinical workplace environment: Evaluating the psychometric properties of the clinical workplace learning NAQ-R scale. Medical Teacher. (2020) 42 (7):813–21., PMID: 32286111 10.1080/0142159X.2020.1746249

[29] Nguyen T Pu C Waits A Tran TD Balhara YPS Huynh QTV. Sources of stress, coping strategies and associated factors among Vietnamese first-year medical students. PloS One. (2024) 19:e0308239. doi: 10.1371/journal.pone.0308239, PMID: 39089322 PMC11290621

[30] Aryuwat P Holmgren J Asp M Radabutr M Lövenmark A. Experiences of nursing students regarding challenges and Support for Resilience during Clinical Education: a qualitative study. Nurs Rep. (2024) 14:1604–20. doi: 10.3390/nursrep14030120, PMID: 39051356 PMC11270303

[31] Ramsland K. The psychology of Death Investigations: Behavioral Analysis for Psychological Autopsy and Criminal Profiling. Boca Raton: CRC Press (2017).

[32] Pal MI Tariq SA Naheed K Ayub A Ahmad E Misbah Z. Comparative analysis of unnatural deaths in Faisalabad during 2018-2022–A raising trend. Prof Med J. (2024) 31:698–703.

[33] Uddin H Hasan MK Cuartas-Alvarez T Castro-Delgado R. Effects of mass casualty incidents on anxiety, depression, and post-traumatic stress disorder among doctors and nurses: a systematic review. Public health. (2024) 234 (2):132–42. doi: 10.1016/j.puhe.2024.06.001, PMID: 39002283

[34] Meeker SA Hahn R Wilt VL Molnar BE. Vicarious traumatization among emergency medical service personnel: A systematic review. Trauma Violence Abuse. (2025) 26(2):15248380251320990. doi: 10.1177/15248380251320990, PMID: 40099545

[35] Kralj A Payne A Holzhauer-Conti O Young J Meiser-Stedman R. Intrusive thoughts and memories in adolescents with major depressive disorder or post-traumatic stress disorder. Br J Clin Psychol. (2024) 63:543–57. doi: 10.1111/bjc.12488, PMID: 38934114

[36] Smaliukienè R Bekesiene S Hoskova-Mayerova S. Emotional resilience for wellbeing and employability: the role of learning and training. Front Psychol. (2024) 15:1379696. doi: 10.3389/fpsyg.2024.1379696, PMID: 38495422 PMC10940539

[37] Bjekić D. Empowering psychological resilience: evaluation of training. In: 10th International Scientific Conference Technics, Informatics and Education-TIE 2024. Kragujevac: Faculty of Technical Sciences Čačak, University of Kragujevac (2024).

